# Synthesis and Spectral Characteristics Investigation of the 2D-2D vdWs Heterostructure Materials

**DOI:** 10.3390/ijms22031246

**Published:** 2021-01-27

**Authors:** Tao Han, Hongxia Liu, Shulong Wang, Shupeng Chen, Kun Yang, Zhandong Li

**Affiliations:** Key Laboratory for Wide-Band Gap Semiconductor Materials and Devices of Education, The School of Microelectronics, Xidian University, Xi’an 710071, China; 15639119745@163.com (T.H.); spchen@xidian.edu.cn (S.C.); kuny2019@163.com (K.Y.); dong2890530@163.com (Z.L.)

**Keywords:** vdWs heterostructure, Raman spectrum, PL spectrum

## Abstract

Due to the attractive optical and electrical properties, van der Waals (vdWs) heterostructures constructed from the different two-dimensional materials have received widespread attention. Here, MoS_2_/h-BN, MoS_2_/graphene, WS_2_/h-BN, and WS_2_/graphene vdWs heterostructures are successfully prepared by the CVD and wet transfer methods. The distribution, Raman and photoluminescence (PL) spectra of the above prepared heterostructure samples can be respectively observed and tested by optical microscopy and Raman spectrometry, which can be used to study their growth mechanisms and optical properties. Meanwhile, the uniformity and composition distribution of heterostructure films can also be analyzed by the Raman and PL spectra. The internal mechanism of Raman and PL spectral changes can be explained by comparing and analyzing the PL and Raman spectra of the junction and non-junction regions between 2D-2D vdWs heterostructure materials, and the effect of laser power on the optical properties of heterostructure materials can also be analyzed. These heterostructure materials exhibit novel and unique optical characteristics at the stacking or junction, which can provide a reliable experimental basis for the preparation of suitable TMDs heterostructure materials with excellent performance.

## 1. Introduction

Nanoscale PN heterostructure research is particularly important as the device size decreases. However, due to the dangling bonds and lattice mismatch, it is difficult for the traditional materials to achieve atomically flat heterostructure interfaces, which limits the development of nanoscale PN heterostructures [1,2,3]. Two-dimensional (2D) layered materials have attracted widespread attention due to their novel and unique electronic and optical properties [4,5]. The discovery of graphene and transition metal dichalcogenide (TMD) materials has brought new opportunities for the development of PN junction components [6]. The layers of 2D materials can be connected by weak van der Waals (vdWs) interactions and there are no surface dangling bonds [7,8,9]. This is conducive to the formation of high-quality vdWs heterostructures, which cannot be limited by the lattice matching conditions [10]. The surface of 2D layered materials are without dangling bonds, which can be combined by vdWs forces. Nanoscale vdWs heterostructures can be prepared by simply stacking 2D materials, which can not only allow studies on some new physical mechanisms, but also realize integrated electronic and optoelectronic devices based on various functions of atomic-level flat heterostructures [11,12,13].

Graphene has the novel physical properties, strong mechanical stability, high mobility and the unique electronic properties of zero-band gap Dirac semimetals, which can make it a basic component of vdWs heterostructures [14,15]. The atoms of hexagonal boron nitride (h-BN) can be bound by strong ionic bonds and there are no dangling bonds on the surface, so its physical and chemical properties are relatively stable. Based on the abovedescribed unique properties, h-BN is widely used as a substrate material [16,17]. Layered TMD materials have become the focus of basic research and technical applications, which is due to their unique crystal structures, a wide range of chemical compositions and their excellent physical and chemical properties [18]. Specific heterostructures can be manufactured by stacking the 2D layered materials with different properties. At this time, the mechanical stability of 2D material can be maintained due to the strong covalent bonding of heterostructure layers and weaker vdWs interactions between the layers can connect different layer materials [19,20,21]. vdWs heterostructures can provide additional degrees of controllable freedom, such as the number of layers and the corners between floors, etc [22]. The energy band structure and electron transport characteristics of systems can be changed by adjusting these degrees of freedom. There are no dangling bonds on the surface of layered materials, so they can be used to design and form various high-quality vdWs heterostructures and novel electronic devices with special functions [23]. Due to the existence of weaker vdWs forces between layers, 2D materials can be easily thinned by a mechanical stripping method. The chemical vapor deposition (CVD) method still has great challenges in the control of film size and number of layers, large-scale heterostructure preparation and free construction, which have become important factors restricting the scale and industrial application of 2D TMDs heterostructure materials [24]. Besides, the in depth study of the excitonic emission, energy band regulation and interlayer coupling basic characteristics of 2D heterostructure materials, which are the basis for the realization of various device applications, has very important scientific significance and practical value.

To solve the difficulties and challenges in the application of 2D-2D vdWs heterostructure materials, vdWs heterostructure materials were prepared by the direct CVD and wet transfer methods in this paper. The resulting vdWs heterostructures have ultra-clean and atomically clear interfaces, and novel physical properties appear when 2D materials with different properties are stacked together, which can provide a multifunctional research platform for the study of interface-related characteristics [25]. In addition, the components material selection, layer thickness and the twist angle between layers can greatly enrich the types of vdWs heterostructure materials [26]. Their optical and electronic properties can be tuned by adjusting their multiple degrees of freedom, which has shown great application prospects in the fields of optoelectronic devices, energy and catalysis.

## 2. Results and Discussion

### 2.1. Optical Micrographs of 2D-2D vdWs Heterostructure Materials

**Figure 1:**
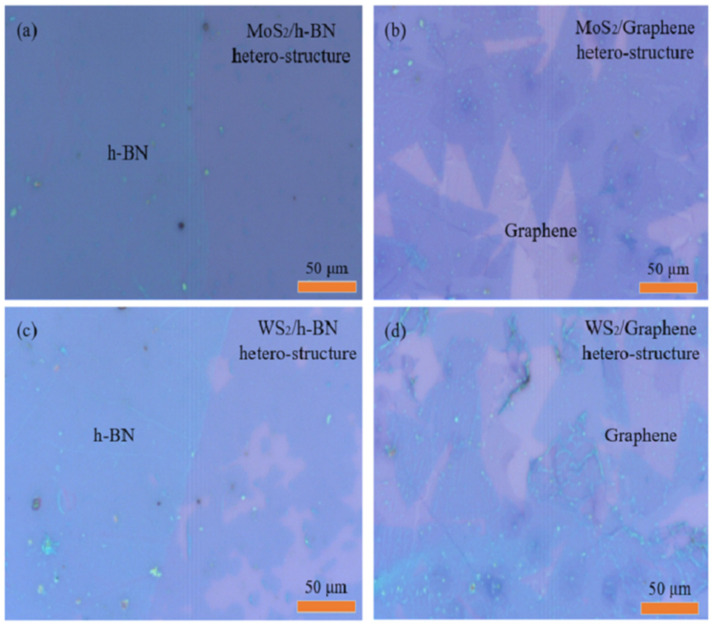
Optical micrograph of (**a**) MoS_2_/h-BN, (**b**) MoS_2_/graphene, (**c**) WS_2_/h-BN, (**d**) WS_2_/graphene heterostructures at different positions on SiO_2_/Si substrate.

The morphology of 2D-2D vdWs heterostructure materials can be observed by optical microscopy. In Figure 1a, a continuous MoS_2_ thin film is formed on a h-BN/SiO_2_/Si substrate by the Frank Van der Merwe mechanism. This is because the surface of h-BN is clean and smooth, which is beneficial to the sulfidation and diffusion of MoS_2_ molecules. The large-sized triangular MoS_2_ materials are evenly distributed on graphene when the CVD growth of MoS_2_ material is completed, and the MoS_2_ and graphene materials on SiO_2_/Si substrate can be well distinguished with the help of contrast as shown in Figure 1b. Figure 1c is the optical microscopy image of WS_2_/h-BN heterostructure at different positions on the SiO_2_/Si substrate. The average size of the WS_2_ material on h-BN/SiO_2_/Si substrate is about 30 µm, the thickness is uniform and the surface is smooth. The edge of the h-BN sheet has many dangling bonds, which can provide nucleation sites for the growth of WS_2_ material. Figure 1d shows a typical optical microscope image of a WS_2_/graphene heterostructure, where the triangle-shaped WS_2_ sheet has an average size of 60 μm. In addition, there is also a stack of small triangles on the large triangle of WS_2_ material, which indicates the existence of multilayer triangular WS_2_ sheets.

### 2.2. The Test Experiment Results of 2D Materials

**Figure 2:**
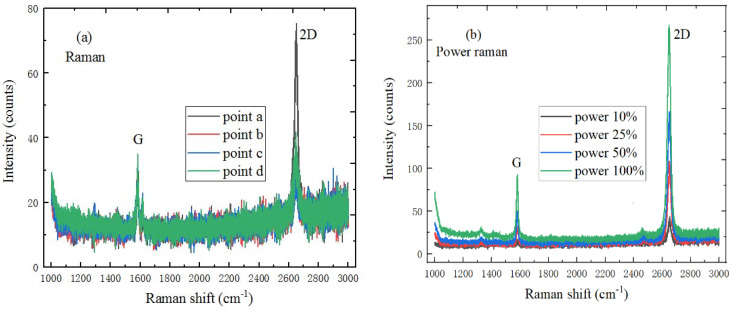
Spectral characteristics of graphene (**a**) Raman spectrum (10 mW, 633 nm), (**b**) Power Raman spectrum (2 mW, 5 mW, 10 mW, 20 mW and 633 nm).

In Figure 2a, graphene material has G and 2D Raman characteristic peaks; the peak position of the G and 2D peaks are respectively located at 1585.9 cm^−1^ and 2631.3 cm^−1^, and the intensity of the 2D peak is stronger than that of the G peak, so the monolayer graphene material has good quality [27]. Figure 2b shows the power Raman spectrum of graphene material, where the peak intensity of the G and 2D peaks increases with the increase of laser power, and the peak position does not change. Meanwhile, the ratio of I_2D_/I_G_ decreases, which is due to the interaction change between graphene material and the underlying SiO_2_/Si substrate.

**Figure 3:**
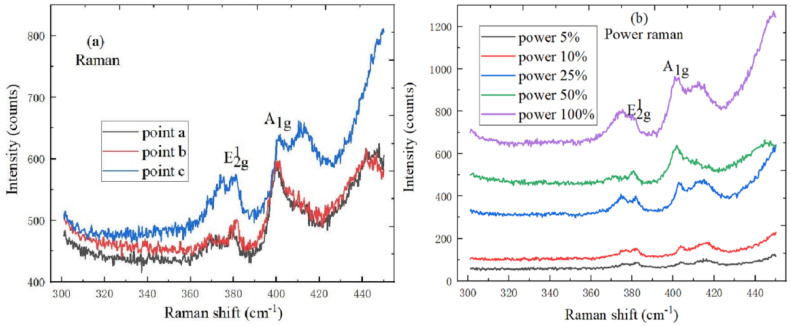
Spectral characteristics of MoS_2_. (**a**) Raman spectrum (10 mW, 633 nm) at different positions, (**b**) power Raman spectrum (1 mW, 2 mW, 5 mW, 10 mW, 20 mW and 633 nm).

Figure 3a shows the Raman spectrum of MoS_2_ at different positions, the lattice vibration of MoS_2_ has the E^1^_2g_ and A_1g_ Raman active characteristic phonon peaks, where the E^1^_2g_ and A_1g_ characteristic peaks were respectively derived from intra-layer lattice and inter-layer lattice vibrations. The two lattice vibration modes change accordingly with the layer number of MoS_2_ material decreases, and the E^1^_2g_ and A_1g_ characteristic peaks are respectively red-shifted and blue-shifted, so the number of MoS_2_ material layers can be determined by the peak position difference. In the Raman spectrum of MoS_2_, the characteristic peaks of E^1^_2g_ mode and A_1g_ mode are respectively located at 382.1 cm^−1^ and 401.4 cm^−1^, and the characteristic peak position difference is 19.3 cm^−1^, which indicates that the MoS_2_ sample is a single layer. Figure 3b is the power Raman spectrum of MoS_2_, the peak intensities of E^1^_2g_ and A_1g_ characteristic peaks accordingly increase with the laser power increases and the peak position of A_1g_ characteristic peaks is blue-shifted to some extent.

**Figure 4:**
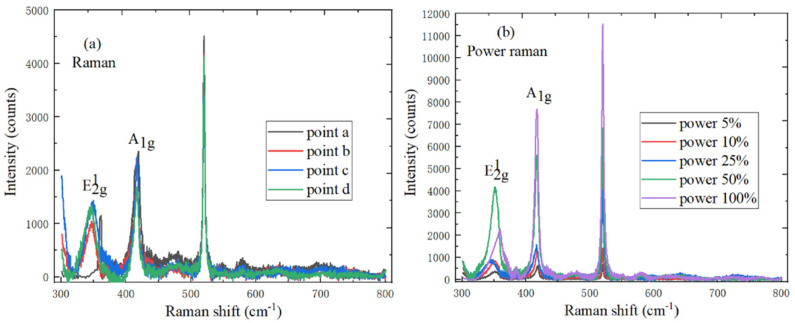
Spectral characteristics of WS_2_ (**a**) Raman spectrum (10 mW, 633 nm) at different positions; (**b**) power Raman spectrum (1 mW, 2 mW, 5 mW, 10 mW, 20 mW and 633 nm).

Figure 4a shows the Raman spectrum of monolayer WS_2_, the E^1^_2g_ and A_1g_ mode characteristic peaks are respectively located at 351.5 cm^−1^ and 416.4 cm^−1^, the peak position difference is 64.9 cm^−1^, and there is no peak near 310 cm^−1^, which indicates the existence of monolayer WS_2_. It can be found by observing Figure 4b that the laser power has a great influence on the peak position and intensity of the E^1^_2g_ vibration characteristic peak and the peak position of A_1g_ vibration mode characteristic peak remains unchanged. This is because the electron concentration changes as the laser power increases, and the increase of electron concentration could cause a reforming of the band gap.

### 2.3. The Test Experiment Results of 2D-2D vdWs Heterostructure Materials

#### 2.3.1. The Characterization of MoS_2_/h-BN Heterostructure

**Figure 5:**
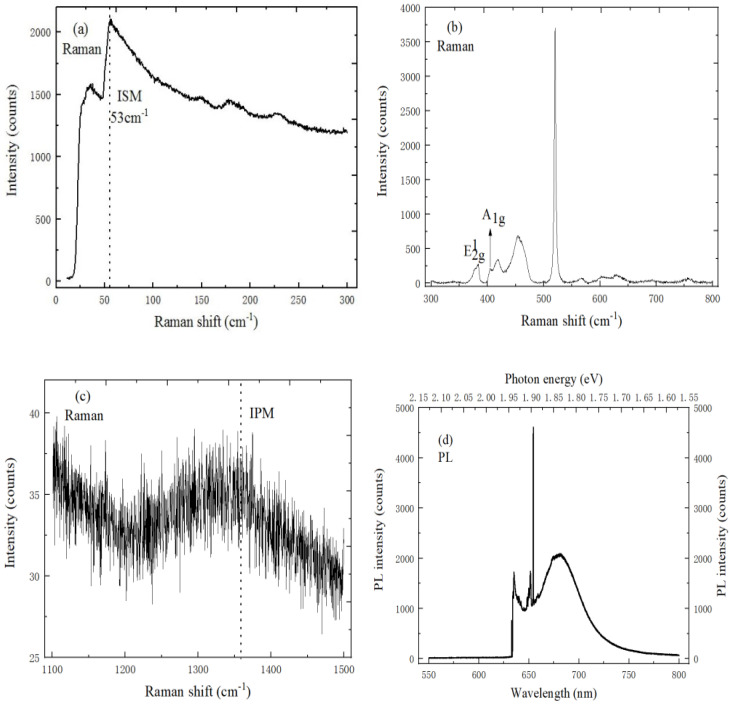
Spectral characteristics of MoS_2_/h-BN heterostructure. (**a**) ISM mode characteristic peak of h-BN (10 mW, 633 nm), (**b**) E^1^_2g_ and A_1g_ mode characteristic peak of MoS_2_ (10 mW, 633 nm), (**c**) IPM mode characteristic peak of h-BN (10 mW, 633 nm), (**d**) PL spectrum of MoS_2_/h-BN heterostructure (20 mW, 633 nm).

In order to match the excitation energy to the PL spectrum exciton peak energy of MoS_2_ material, the resonance Raman scattering of MoS_2_/h-BN heterostructure material can be studied by analyzing the band structure and exciton transition. Figure 5a,c respectively represent the low-frequency ISM mode and high-frequency IPM mode characteristic peaks of h-BN, where the corresponding peaks are located at 53 cm^−1^ and 1358 cm^−1^.

At the same time, the full width at half maxima (FWHM) of the IPM mode characteristic peak is much larger than that of the ISM mode. Figure 5b shows the E^1^_2g_ and A_1g_ mode characteristic peaks of MoS_2_ on a h-BN substrate. The in-plane vibration E^1^_2g_ mode characteristic peak and out-plane vibration A_1g_ mode characteristic peak are respectively located at 384.6 cm^−1^ and 404.2 cm^−1^, and the frequency difference is 19.6 cm^−1^. Compared with the Raman spectrum of MoS_2_ on SiO_2_/Si substrate, the characteristic peak frequencies of E^1^_2g_ mode and A_1g_ mode are respectively blue-shifted by 2.5 cm^−1^ and 2.8 cm^−1^ on h-BN/SiO_2_/Si substrate, and the substrate effect can explain the blue shift of the Raman peak on h-BN. Therefore, Raman spectroscopy at room temperature showed that the h-BN substrate can reduce the local strain and injected impurities into MoS_2_. Besides, the PL spectrum can be fitted by the Lorentz function, as shown in Figure 5d. The strongest PL peak position of MoS_2_ is at 681 nm, and the corresponding energy electron volt band gap is 1.82 eV, which is consistent with the band gap of monolayer MoS_2_. The PL exciton emission of MoS_2_/h-BN heterostructure material is two times larger than that of MoS_2_ material, and the FWHM is 26.39 nm, which is narrower than that of MoS_2_ material. Therefore, MoS_2_/h-BN heterostructure material has a tighter interlayer contact and lower charged impurities levels.

#### 2.3.2. The Characterization of MoS_2_/Graphene Heterostructure

When monolayer MoS_2_ material is grown directly on graphene to form a MoS_2_/graphene heterostructure, their lattice vibrations would inevitably affect each other. In a MoS_2_/graphene heterostructure, the work function of graphene is smaller than that of MoS_2_ material, which is beneficial to the formation of contacts.

**Figure 6:**
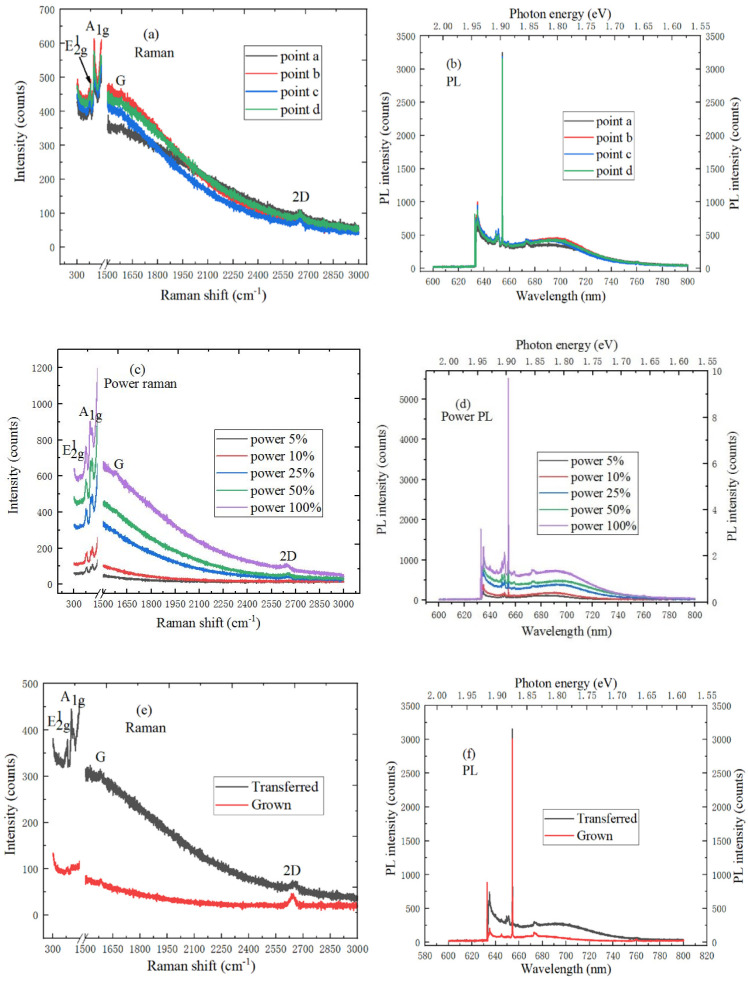
Spectral characteristics of MoS_2_/graphene heterostructure. (**a**) Raman spectrum (10 mW, 633 nm) and (**b**) PL spectrum (20 mW, 633 nm) at different positions; (**c**) power Raman spectrum (1 mW, 2 mW, 5 mW, 10 mW, 20 mW and 633 nm), (**d**) power PL spectrum (1 mW, 2 mW, 5 mW, 10 mW, 20 mW and 633 nm); (**e**) Raman spectrum (10 mW, 633 nm) and (**f**) PL spectrum (20 mW, 633 nm) comparison between MoS_2_ sample directly grown on a graphene and MoS_2_ sample transferred on a graphene.

In Figure 6a, the E^1^_2g_ and A_1g_ modes characteristic peaks of MoS_2_ material and the G and 2D peaks of graphene appear in the Raman spectrum, which indicates the presence of MoS_2_/graphene heterostructures. Among them, the E^1^_2g_ and A_1g_ mode characteristic peak positions of MoS_2_ material are respectively located at 382.6 cm^−1^ and 400.3 cm^−1^, and the G and 2D peak positions of graphene are located at 1602.5 cm^−1^ and 2689.7 cm^−1^, respectively. The G and 2D peak positions of the MoS_2_/graphene heterostructure shift to larges wavenumbers compared with the spectrum of intrinsic graphene, the the G and 2D peaks respectively change from 1585.9 cm^−1^ and 2631.3 cm^−1^ to 1602.5 cm^−1^ and 2689.7 cm^−1^. The reason is that the hole concentration becomes smaller and the electrons from MoS_2_ material can be transferred to the graphene. Besides, the E^1^_2g_ and A_1g_ mode characteristic peaks of MoS_2_/graphene heter-structure are respectively red-shifted and blue-shifted by 0.5 cm^−1^ and 1.1 cm^−1^ compared with the spectrum of monolayer MoS_2_, which is due to the Van der Waals force interactions between MoS_2_ and graphene. Figure 6b shows the PL spectrum of MoS_2_/graphene heterostructure material at different positions. The strongest PL spectrum peak position of MoS_2_/graphene heterostructure is at 674 nm, and the corresponding electron volt energy is 1.84 eV. The peak position of strongest PL spectrum shows the blue shift, graphene has the influence on the Raman spectrum of MoS_2_ material. The energy band of MoS_2_ material bends downward, and the Schottky barrier is lower when the Fermi level of graphene and MoS_2_ materials are aligned. It can be found by observing Figure 6c that the intensity of characteristic peaks also increases with the laser power increases, and the positions of characteristic peaks shift. This is because as the test temperature of MoS_2_/graphene heterostructure becomes higher, it will cause the spectrum to change. The rise of 2D and G peaks position is related to the effective interlayer coupling of MoS_2_ nanosheets, and the strain effect, which is caused by the high temperature heating. Besides, due to the PL phenomenon of MoS_2_, the background Raman signal increases with the wavenumber increase. As shown in Figure 6d, the peak intensity of strongest PL spectrum increases with the increase of laser power, and the corresponding peak positions shift. This is because the spectrum changes with temperature. In addition, the 2D peak of MoS_2_/graphene heterostructure is superimposed on B exciton, and the peak position has the blue shift, which can be explained by the stacking effect [28]. Figure 6e shows the Raman spectrum comparison between MoS_2_ sample directly grown on a graphene and MoS_2_ sample transferred on a graphene substrate. The E^1^_2g_ peak position of monolayer MoS_2_ material is blue-shifted by 1.3 cm^−1^ after the transfer, and the A_1g_ peak position only changes slightly. There is a certain tension effect when monolayer MoS_2_ material is grown on the SiO_2_/Si substrate by CVD. After the transfer, the A1 peak position of PL spectrum in MoS_2_ sample has a blue shift about 40 meV, as shown in Figure 6f. The peak intensity of PL spectrum in MoS_2_ sample transferred on a graphene is higher than that of MoS_2_ sample directly grown on a graphene. The reason is that the tension of the sample is effectively released while using PMMA to transfer MoS_2_ material to the new SiO_2_/Si substrate.

**Figure 7:**
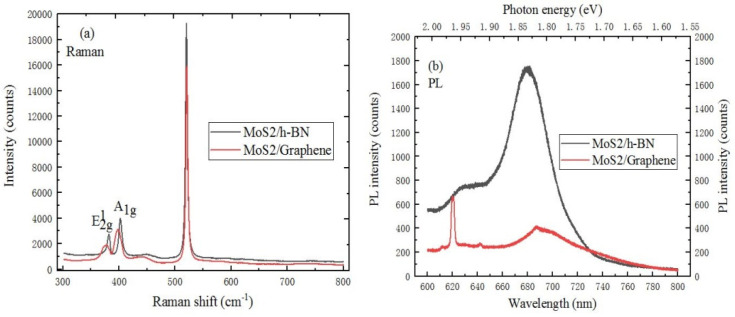
Spectral characteristics of MoS_2_ on h-BN and graphene substrate (**a**) Raman spectrum (10 mW, 633 nm), (**b**) PL spectrum (20 mW, 633 nm).

A 633 nm red laser was used to compare and analyze the spectral characteristics of MoS_2_ on h-BN and graphene substrates. As we all know, the MoS_2_ material has a suitable and adjustable band gap, strong exciton interaction, unique nonlinear effects and a novel spin valley effect, which has the great application potential in the fields of integrated electronic chips, photoelectric conversion and the energy environment. In Figure 7a, the E^1^_2g_ and A_1g_ mode characteristic peaks of MoS_2_/graphene heterostructure are blue-shifted relative to the MoS_2_/h-BN heterostructure material. The reason is that the graphene substrate has the effect on the Raman spectrum of MoS_2_. Vertical heterostructures can be formed by the van der Waals force between the upper and lower layers, which would lead to a strong quantum confinement effect. This can not only change the electronic band and optical properties of MoS_2_ material, but also give it more excellent chemical activity. It can be found from Figure 7b that the strongest PL peak position of MoS_2_/graphene heterostructure material is red-shifted compared with MoS_2_/h-BN heterostructure, and the peak intensity is weaker than that of MoS_2_/h-BN heterostructure. This is because graphene has the weakening effect on the fluorescence of MoS_2_. Besides, the exciton absorption peak of PL spectrum in MoS_2_/graphene heterostructure is located at 621 nm. The change of thickness and the abundance of heterostructures can further produce and broaden the new characteristics and application research of MoS_2_ material.

#### 2.3.3. The Characterization of WS_2_/hBN Heterostructure

**Figure 8:**
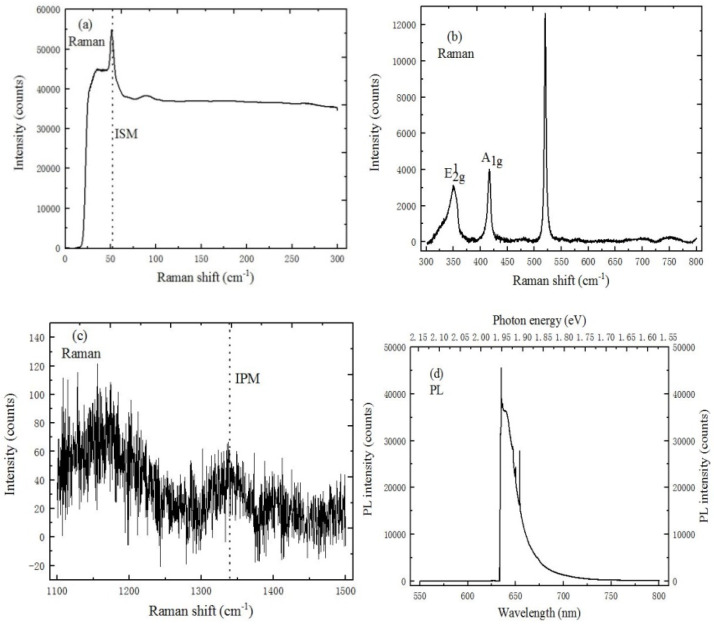
Spectral characteristics of WS_2_/h-BN heterostructure. (**a**) ISM mode characteristic peak of h-BN (10 mW, 633 nm), (**b**) E^1^_2g_ and A_1g_ mode characteristic peaks of WS_2_ (10 mW, 633 nm), (**c**) IPM mode characteristic peak of h-BN (10 mW, 633 nm), (**d**) PL spectrum of WS_2_/h-BN heterostructure (20 mW, 633 nm).

Figure 8a,c respectively show the ISM and IPM mode characteristic peaks of WS_2_/h-BN heterostructure materials where the corresponding peak positions are located at 52 cm^−1^ and 1346 cm^−1^, respectively.

Compared to the spectrum of MoS_2_/h-BN heterostructure, the peak position of ISM and IPM mode characteristic peaks are blue-shifted. The reason is that WS_2_ material has an influence on the spectrum of h-BN. It can be clearly observed from Figure 8b that the E^1^_2g_ and A_1g_ Raman activation mode peak positions of WS_2_ are respectively located at 354.8 cm^−1^ and 419.4 cm^−1^, and the peak spacing is 64.6 cm^−1^, which corresponds to the peak spacing of monolayer WS_2_ on SiO_2_/Si substrate. The peak position of WS_2_/h-BN heterostructure is different from that of WS_2_ on SiO_2_/Si substrate. The reason is that the van der Waals interaction between layers, and the effective transfer between dielectric screen of long-distance Coulomb interactions. In Figure 8d, the peak position of the strong peak is located at 1.95 eV. Meanwhile, the FWHM of PL emission peak is less than that of WS_2_ material. The FWHM of PL emission peak is related to the exciton lifetime and interface quality, which can be attributed to the extension of high crystallinity WS_2_ material, and the clean heterojunction interface.

#### 2.3.4. The Characterization of WS_2_/Graphene Heterostructure

**Figure 9:**
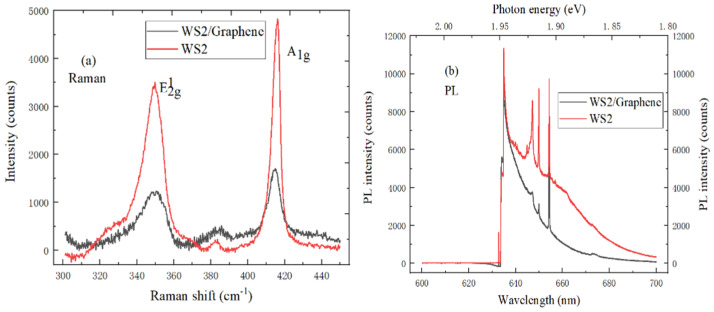
(**a**) Raman spectrum (10 mW, 633 nm), (**b**) PL spectrum (20 mW, 633 nm) characteristics comparison of WS_2_ and WS_2_/graphene heterostructure.

Raman spectroscopy is used to evaluate the crystal quality and film thickness of 2D materials. The Raman spectrum contrast analysis of WS_2_ and WS_2_/graphene heterostructures is shown in Figure 9a, the peak position of A_1g_ mode characteristic peak of WS_2_/graphene heterostructure shifts to the short wavelength compared with the Raman spectrum of WS_2_, and the peak position of A_1g_ characteristic peak has the blue shift. And the E^1^_2g_ and A_1g_ mode characteristic peak intensity of WS_2_/graphene heterostructure is higher than that of WS_2_.

There is the sharp Raman characteristic peak, which indicates that WS_2_/graphene heterostructure film has excellent crystallinity. In addition, monolayer WS_2_ and multilayer WS_2_ materials are respectively the direct bandgap semiconductor and indirect semiconductor, so the PL spectroscopy can also be used to identify the number of layers. In Figure 9b, the PL spectrum of WS_2_ and WS_2_/graphene heterostructure both show the strongest PL emission peak is located at 634 nm, and the band gap is about 1.94 eV, which indicates that it is consistent with a mechanically peeled monolayer WS_2_. The PL intensity of WS_2_/graphene heterostructure is weaker than that of monolayer WS_2_. This is because the work function between graphene and WS_2_ material does not match, thus forming the internal field, which would cause the photoelectrons from WS_2_ can transfer to graphene, and leave holes in WS_2_.

**Figure 10:**
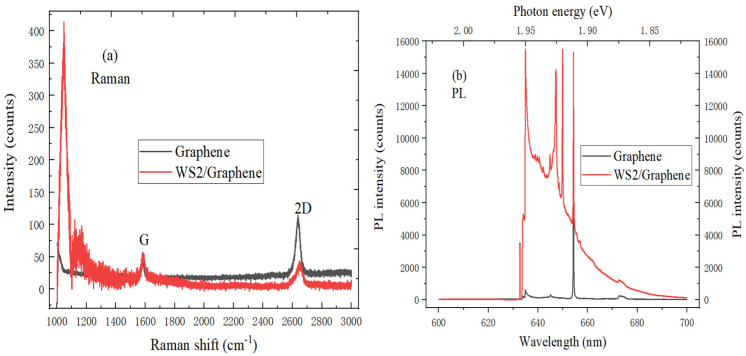
(**a**) Raman spectrum (10 mW, 633 nm), (**b**) PL spectrum (20 mW, 633 nm) comparison of graphene and WS_2_/graphene heterostructure.

In Figure 10a, the typical G peak and 2D peak of intrinsic graphene were respectively located at 1583.5 cm^−1^ and 2667.5 cm^−1^, and the intensity of 2D peak is almost twice that of D peak, which indicates the presence of monolayer graphene. The D peak appears in the Raman spectrum of graphene material when the growth of WS_2_ material is completed. Meanwhile, the peak position of 2D peak shifts downward, which is due to the effective interlayer coupling and strain effect. As shown in Figure 10b, the background Raman signal of PL spectrum increases with the increase of wavenumber, which is attributed to the PL phenomenon of WS_2_. At the same time, the internal field can be formed, which is due to the work function mismatch between graphene and WS_2_.

**Figure 11:**
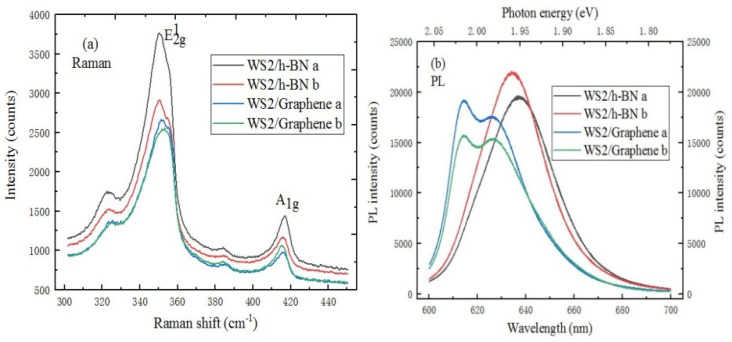
(**a**) Raman spectrum (10 mW, 633 nm), (**b**) PL spectrum (20 mW, 633 nm) comparison of WS_2_ on h-BN/SiO_2_/Si or graphene/SiO_2_/Si substrate.

A Raman spectrum characteristics comparison of WS_2_/h-BN and WS_2_/graphene heterostructure is shown in Figure 11a. Compared to the spectrum of WS_2_/h-BN heterostructure, the E^1^_2g_ and A_1g_ mode characteristic peaks of WS_2_/graphene heterostructure are respectively red-shifted and blue-shifted. The reason is that graphene material has a influence on the spectrum of WS_2_ material. In Figure 11b, the strongest PL peak position of WS_2_/graphene heterostructure is blue shifted. The difference of peak position shift can be attributed to the influence of different substrates and the change of interaction.

## 3. Materials and Methods

### 3.1. Preparation of Graphene and h-BN Materials

First, the copper foil substrate was used in the growth process of graphene and h-BN materials by the CVD growth process. Before the growth of graphene and h-BN films on copper foil substrate, it is necessary to immerse the copper substrate in hydrochloric acid for 15 min to remove the local surface oxides. Finally, the copper foil substrate can be rinsed with the acetone and isopropanol solution.

**Figure 12:**
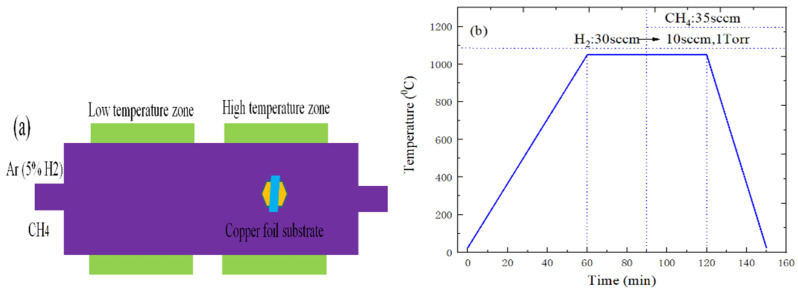
(**a**) the preparation schematic diagram of graphene; (**b**) temperature change curve of graphene.

Figure 12a shows the preparation schematic diagram of graphene. First, the cleaned copper substrate was placed in a CVD furnace. Then, the chamber was evacuated to the vacuum pressure of 1 mTorr to remove any harmful gases. Finally, the chamber of tube furnace can restore the atmospheric pressure by filling the chamber with dilute argon gas diluted with 5% hydrogen. The following describes the main growth processes of graphene material. First, the copper foil substrate of tube furnace was annealed, the maximum temperature and annealing time were set to 1050 °C and 30 min during the annealing process, and 30 sccm H_2_ was also punched. Next, the tube furnace was charged with 35 sccm CH_4_, while the flow rate of H_2_ was adjusted to 10 sccm, and the growth time of graphene was 30 min, as shown in Figure 12b. Finally, the tube furnace is cooled to room temperature by the forced cooling, which can also prevent the CH_4_ gas from flowing into the tube furnace.

**Figure 13:**
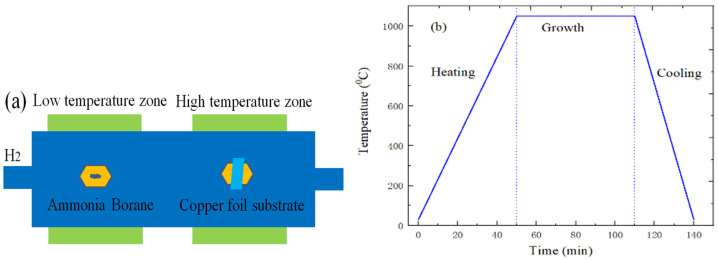
(**a**) the growth schematic diagram of h-BN; (**b**) CVD growth temperature change curve of h-BN.

Figure 13a is the preparation schematic diagram of h-BN on the copper foil substrate by CVD method. First, the tube furnace was heated to 1050 °C, and the copper foil substrate was annealed and recrystallized at the temperature for 30 min. Then, while entering the growth stage, the ammonia borane can decompose to form the hydrogen (H_2_ gas), aminoborane polymer (aminoborane) and borazine under the action of heating belt [29]. At the same time, boron azepine enters the high temperature heating area of tube furnace under the carrier gas action of 10 sccm H_2_. Next, the borazine can dehydrogenated again due to the catalytic effect of the copper foil metal, and the B and N atoms combined to form h-BN on SiO_2_/Si substrate. Finally, the power supply of heating belt and furnace is quickly cut off when the h-BN growth reaction is completed, and the tube furnace enters the cooling stage. The specific temperature change curve is shown in Figure 13b.

### 3.2. The Transfer of Graphene or h-BN

The graphene or h-BN material grown on Cu foil substrate by CVD method was transferred to SiO_2_/Si substrate, which can be achieved by the PMMA transfer method. First, the 500 nm PMMA layer was spin-coated on the graphene or h-BN/copper foil substrate, and the PMMA film was cured at 120 °C for 3 min when the spin coating was completed. Then, the copper foil substrate was etched by 1M FeCl_3_ solution. Next, the PMMA/graphene or PMMA/h-BN layers were rinsed several times in deionized water, which can be transferred to the SiO_2_/Si substrate. Subsequently, the sample was dried in the N_2_ gas environment for 12 h to make the graphene or h-BN and SiO_2_/Si substrate adhere more closely. Immediately after, the PMMA film was washed away in the acetone solution.

### 3.3. The Preparation of WS_2_ or MoS_2_ Material

The purpose of oxygen plasma treatment is to make the SiO_2_/Si substrate hydrophilic, and the treatment is 2 min, which can help the transfer of graphene or h-BN film and the growth of MoS_2_ or WS_2_ materials [30]. The SiO_2_/Si substrate was annealed at 400 °C for 8 h when graphene or h-BN thin film was transferred to SiO_2_/Si substrate. Meanwhile, the Ar gas with 5% H_2_ was continuously provided to remove residues during the transfer process.

**Figure 14:**
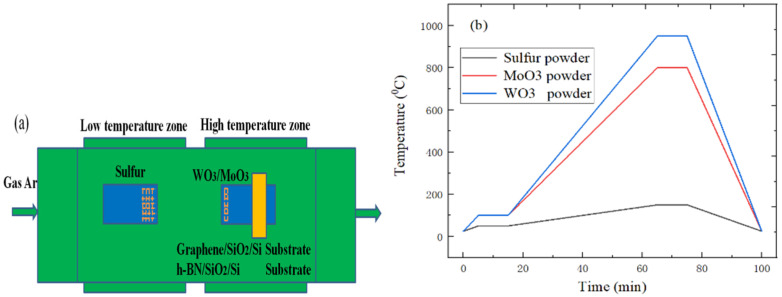
(**a**) Experimental schematic diagram of WS_2_ and MoS_2_ growth; (**b**) The relationship between temperature and time in different heating zones.

The WS_2_ or MoS_2_ materials were grown on the graphene/SiO_2_/Si or h-BN/SiO_2_/Si substrate in the tube furnace, and the SiO_2_/Si samples coated with graphene or h-BN materials were used as the substrates. The quartz boat containing 200 mg sulfur powder was placed upstream of the tube furnace, which is the low-temperature heating zone. At the same time, the graphene/SiO_2_/Si or h-BN/SiO_2_/Si substrate were placed upside down on the quartz boat containing WO_3_ or MoO_3_ powder, which is located in the high temperature heating area of tube furnace, as shown in Figure 14a. It can be found from Figure 14b that the temperature of sulfur powder and MoO_3_ powder (or WO_3_ powder) respectively rises to 50 °C, 100 °C, 100 °C within 5 min, and maintains at this temperature for 10 min. Next, the temperature of sulfur powder and MoO_3_ powder (or WO_3_ powder) can respectively reach the sublimation temperature of 150 °C, 800 °C and 950 °C within 50 min, and the growth temperature is maintained for 10 min. Finally, the tube furnace was naturally cooled to room temperature.

### 3.4. The Test Characterization of Heterojunction Materials

Raman spectrometry can confirm the molecular structure by the Raman shift. The incident light is scattered when a certain wavelength of laser is irradiated on the sample surface, and the frequency of most scattered light does not change, which is the Rayleigh scattering. The small part scattering of scattered light frequency is referred to as Raman scattering, and the frequency difference between scattered light and incident light is called Raman shift. Raman spectroscopy is used to monitor the crystal quality, polymorphism, doping, defects, strain, disorder, chemical modification and relative orientation of 2D materials. Besides, Raman analysis has the high spatial resolution, the fast and easy spectral imaging, which is suitable for identifying the characteristics and structure of various materials [31]. The photoluminescence (PL) spectrum is that the sample can obtain the energy while being irradiated by an external light source, which can produce the excitation light emission phenomenon. The internal information of material samples can be obtained by the research and analysis of PL spectroscopy, which can guide the analysis of sample quality, defect distribution information.

The distribution of heterostructure samples on the substrate surface can be observed by optical microscopy, which can test and characterize the aboveprepared heterostructure materials. The spatial resolution depends on the choice of excitation laser to a certain extent, and the excitation wavelength can be optimized based on the characteristics of the sample. Meanwhile, a Raman spectrometer (LabRam HR Evolution, HORIBA Jobin Yvon, Paris, France) with a 633 nm laser red light was used to measure and obtain the single-point Raman and PL spectrum of the heterostructure materials, and the surface uniformity and composition distribution of heterostructure film were analyzed by Raman and fluorescence surface scanning. The surface morphology of heterostructure materials can be observed by optical microscopy. For the Raman and PL spectrum tests, the corresponding laser power is respectively fixed at 10 mW and 20 mW, the laser spot diameter is 1 μm, and the objective magnification of microscope is 10. The Raman data of characteristic peaks were analyzed by the classical least squares (CLS) fitting.

## 4. Conclusions

MoS_2_/h-BN, MoS_2_/graphene, WS_2_/h-BN and WS_2_/graphene van der Waals heterostructures were successfully prepared in this paper. The growth mechanism and optical characteristics of heterostructure materials can be studied by optical contrast microscopy and Raman PL spectroscopy, which can obtain the spectral characteristics of heterostructure materials. These heterostructures exhibit the novel and unique optical properties at the stacking or junction of several materials. The Raman and PL spectroscopy models are complementary and can describe the temperature-dependent relationships between interlayer coupling and physical origin. Meanwhile, the electron-phonon coupling and vdW interaction can be enhanced by reducing the local strain and charged impurities. Compared with the Raman spectrum of MoS_2_ on SiO_2_/Si substrate, the characteristic peak frequencies of E^1^_2g_ mode and A_1g_ mode are blue-shifted by 2.5 cm^−1^ and 2.8 cm^−1^ respectively on a h-BN/SiO_2_/Si substrate, and the E^1^_2g_ and A_1g_ mode characteristic peaks of MoS_2_/graphene heterostructure are respectively red-shifted and blue-shifted by 0.5 cm^−1^ and 1.1 cm^−1^. The G and 2D peak positions of MoS_2_/graphene heterostructure shift to the large wavenumber compared with the spectrum of intrinsic graphene. Besides, E^1^_2g_ and A_1g_ mode characteristic peaks of MoS_2_/graphene heterostructure are blue-shifted compared with MoS_2_/h-BN heterostructure, and the strongest peak position of PL spectrum is red-shifted. The crystal orientation between WS_2_ or MoS_2_ growth and hBN or a graphene substrate is significantly different, which is due to the interlayer Coulomb interactions. Our research can effectively improve the interlayer coupling basic understanding of heterostructures and temperature-dependent Raman and PL spectra were used to explore the optical properties of vdWs heterostructures. Therefore, nanoscale vdW heterostructures have the great application prospects in the fields of nanoelectronics and optoelectronics.

## Data Availability

No new data were created or analyzed in this study. Data sharing is not applicable to this article.
